# Physiological correlates of a simple saccadic-decision task to extended objects in superior colliculus

**DOI:** 10.1016/j.isci.2025.113179

**Published:** 2025-07-23

**Authors:** Baptiste Caziot, Bonnie Cooper, Mark R. Harwood, Robert M. McPeek

**Affiliations:** 1 Graduate Center for Vision Research, Department of Biological and Vision Sciences, SUNY College of Optometry, 33 West 42^nd^ Street, New York, NY 10036, USA; 2 Neurophysics Department, Philipps-Universität Marburg, 8A Karl-von-Frisch-Straße, 35043 Marburg, Germany; 3 Center for Mind, Brain and Behavior (CMBB), Philipps-Universität Marburg and Justus–Liebig–Universität Gießen, Gießen, Germany; 4 Department of Biology, City College of New York, New York, NY 10031, USA; 5 Department of Psychology and Human Development, University of East London, E15 4LZ London, UK

**Keywords:** Natural sciences, Biological sciences, Neuroscience, Systems neuroscience, Sensory neuroscience

## Abstract

Saccadic eye movements direct gaze to objects of interest. Human studies show that saccade initiation latency depends on the size of the saccade target (the “size-latency effect”), perhaps reflecting a tradeoff between the cost of making a saccade and the expected information gain. Here, we investigated the neuronal correlates of the size-latency effect in macaque superior colliculus (SC). Analysis of saccade latencies within a stochastic accumulator framework predicted a steeper increase in activity for smaller targets compared to larger ones, and, surprisingly, an increase in saccade initiation threshold for smaller targets. We found that SC activity is in close agreement with these predictions. We also found evidence that these effects may be a consequence of the visual responses of SC neurons to targets of different sizes. The results shed new light on the sources of delay within the saccadic system, a system that we heavily depend upon for visuo-motor tasks.

## Highlights

• Saccadic latencies are strongly modulated by targets size
• Modeling predicts higher accumulation rates and decision thresholds for smaller targets
• Build-up rate and peak of saccade-related SC activity agree with these predictions
• These effects are most likely caused by the initial visual responses of the same cells

## Graphical abstract

**Figure:**
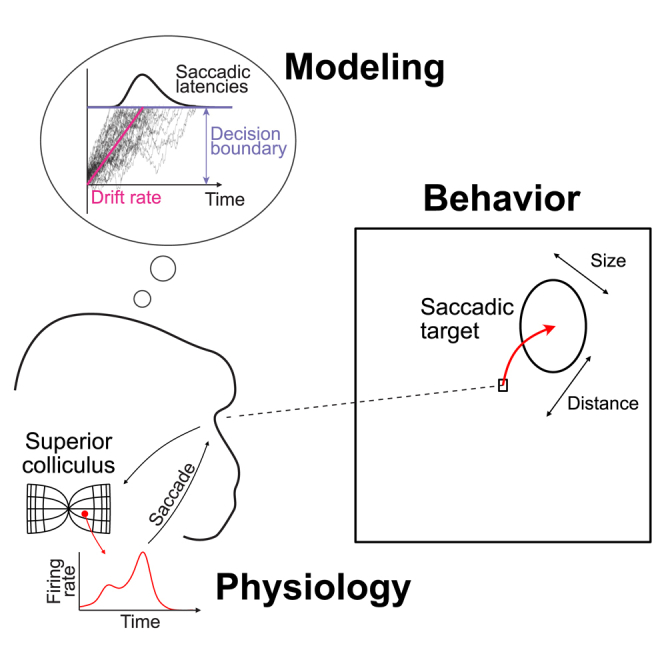

## Introduction

Because vision is most accurate at the fovea, humans constantly move their eyes to refoveate the visual scene, mostly through saccadic eye movements. Although these eye movements are fast and accurate, it has long been observed that they have a surprisingly long initiation latency.^1^ This initiation delay is thought to reflect a decision-making process in which an observer accumulates evidence in favor of making an eye movement. Saccadic eye movements are costly, both physiologically and functionally,^2^^,^^3^ thus this saccadic decision-making probably reflects a compromise between visual acuity gained from orienting the fovea to a new location and the cost for moving the fovea. These decisions are among the most common in everyday life, and yet the link between function and decision signal remains poorly understood.

Multiple factors modulating saccade latencies have been well studied, including stimulus contrast,^4^ inhibition of return,^5^ or the gap effect,^6^^,^^7^^,^^8^^,^^9^ and the neural bases of these factors have been characterized.^10^^,^^11^^,^^12^^,^^13^^,^^14^^,^^15^ Target eccentricity is also known to affect latencies, with a minimum for eccentricities between 3 and 10 deg, steeply rising at shorter eccentricities^16^^,^^17^^,^^18^^,^^19^^,^^20^ and more gradually rising at larger eccentricities.^19^^,^^21^ The neural basis for the dramatic increase in latency for small eccentricity saccade targets has been ascribed either to a fixation zone around the foveal representation in the superior colliculus (SC),^22^ a key structure for saccade generation, or an equilibrium between neurons in the SC.^23^

Unlike arm movements, early studies reported small and inconsistent effects of target size on the kinematics of saccades and their reaction times.^24^^,^^25^^,^^26^^,^^27^ However, later studies found strong effects of target size on saccade latency,^28^^,^^29^^,^^30^^,^^31^ provided that the target sizes are relatively large (larger than the target sizes used by early studies). This effect was named the size-latency effect.^28^^,^^29^^,^^30^^,^^31^ Furthermore, Harwood et al.^28^ found that saccadic latencies are modulated by both the size of a target and its eccentricity, and that they are best explained by the ratio of the two. Therefore, the effects of target size and eccentricity form a continuum where saccadic latencies are modulated by the size of the target relative to its eccentricity.

Here, we first establish that the size-latency effect exists in monkeys, as it does in humans, and go on to investigate the involvement of visuomotor neurons in SC in this effect. To do this, we fit a stochastic accumulator decision model to monkeys’ saccade-latency data to infer the values of putative decision-related variables. We then compare the activity of cells in the intermediate layers of the SC to these predicted variables, finding that movement-related activity in the SC closely matches the predictions from the accumulator model. Finally, we show that these effects are most likely caused by the visual responses of the same neurons.

## Results

### Behavioral results

**Figure 1:**
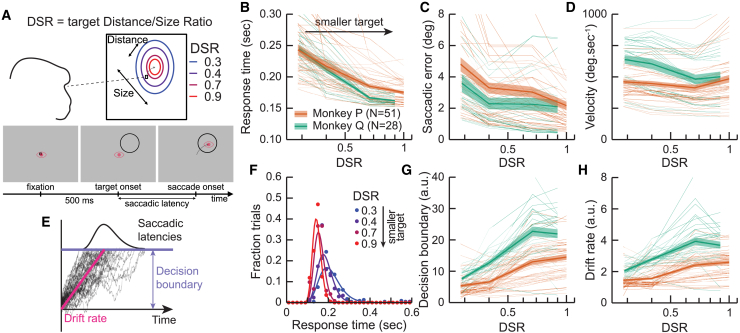
Behavioral results (A) Top: monkeys were trained to make saccades to targets with different Distance-to-Size Ratios (DSRs). A higher DSR corresponds to a smaller and/or more distant target. Only one target was presented on each trial. Bottom: time course of a trial: 500 ms after fixation onset, the ring target was displayed centered on the cell’s preferred saccadic location. Saccadic latency corresponds to the time between target onset and the onset of the saccadic eye-movement. (B) Median saccadic latency as a function of DSR for both animals (green and orange). Thin lines correspond to individual sessions, thick lines to mean across sessions and shaded area to standard errors. (C) Mean saccadic error as a function of DSR for both animals. (D) Peak saccadic velocity as a function of DSR for both animals. (E) Saccadic latencies were modeled as the accumulation of a noisy signal with a drift-rate toward a decision boundary. Saccades are triggered when the decision boundary is crossed, predicting an inverse-Gaussian distribution of saccadic latencies. (F) Example of distributions of saccadic latencies as a function of DSR (blue to red) for a single session. Dots are fraction of trials within 40 ms bins and lines are model fits. (G) Predicted decision boundaries as a function of DSR. (H) Predicted drift-rate as a function of DSR.

We trained 2 monkeys (*Macaca mulatta*) to perform saccadic eye movements toward a large ring target. After a cell exhibiting movement-related activity was isolated, we used a delayed-saccade task to map the preferred saccadic direction and amplitude associated with the cell, and then displayed ring-shaped targets centered at the cell’s preferred location (see Figure S1 for a distribution of cells preferred saccadic locations). Ring sizes were varied from trial-to-trial such that the distance-to-size ratio (DSR = Distance/Size, with distance the eccentricity of the target) had one of 4 values ranging between approximately 0.3 and 1.0. Since the target’s DSR corresponds to the target distance (eccentricity) divided by the target size, a DSR value of 1 corresponds to a target whose size equals its eccentricity. A DSR lower than 0.5 corresponds to a target more than twice as large as it is eccentric, and therefore includes the fovea within its limits (see Figure 1A). Monkeys were rewarded for making a saccade to the center of the rings. Note that here, for a given cell, the monkeys always performed the same saccadic eye movement on every trial, and we elicited the size-latency effect by modulating that size of the target from trial to trial. The eccentricity of the target was selected to correspond to each cell’s preferred eccentricity.^28^

The size of the targets modulated behavior in several ways. Figure 1B plots median saccadic latencies as a function of DSR across sessions for both monkeys. Both monkeys exhibited a significant increase of their saccadic latencies with target size (resampling, see STAR Methods, *p* < 0.001), establishing a strong size-latency effect in monkeys (see Figure S2 for latencies plotted as a function of target size and distance). Saccadic latencies ranged from almost 250 ms for the largest targets to only about 150 ms for the smallest targets. Figure 1C plots mean saccadic error (distance between the eye landing point and the center of the ring target) as a function of DSR. Saccadic errors significantly decreased with decreasing target size for both animals (resampling, *p* < 0.001). Thus, smaller targets are associated with shorter initiation times and higher precision, the opposite of a speed-accuracy tradeoff. Furthermore, saccadic latencies tend to increase slightly with saccadic amplitude (see Figure S3), excluding the possibility that this effect is simply a byproduct of increased saccadic amplitudes. Finally, Figure 1D plots the mean saccade peak velocity as a function of DSR. Peak velocity significantly decreased with smaller targets for monkey Q (resampling, *p* < 0.001), from approximately 640 to 500 deg.sec^−1^, but remained stable at approximately 430 deg.sec^−1^ for monkey P (resampling, *p* = 0.47). Therefore, the size-latency effect caused, as in humans,^28^^,^^29^^,^^30^^,^^31^ a dramatic increase in saccadic latencies for larger targets, as well as larger saccade endpoint spread. In one animal, but not in the other, it was also accompanied by a slight increase in saccade peak velocities for larger targets.

We modeled saccadic decisions as an accumulation of a noisy signal toward a decision boundary (see Figure 1E). This class of models is commonly used to model decision time.^32^ We assumed the noisy signal to be a Wiener process of mean μ > 0 and fixed noise σ = 1 (see Figure S4 for a different model leading to similar conclusions). The distribution of crossing times is given by an inverse Gaussian distribution (see STAR Methods). This model corresponds to the continuous limit of Wald’s sequential probability ratio test against one-sided alternatives,^33^ that is where one is preoccupied only with detecting the presence of a target at a fixed ⍺-error rate (false positives). One-sidedness is warranted here, because the alternative hypothesis would correspond to the animals deciding never to move their eyes.

We fitted inverse Gaussian distributions to the distribution of saccadic latencies using maximum likelihood estimation. Figure 1F shows an example of saccadic latency distributions as a function of DSR (red to blue) for 1 recording session. Lines show that inverse Gaussian distributions fit the data well (see Figure S5). From the model fit, we can predict the information accumulation rate (drift rate) and height of the decision boundary. Figures 1G and 1H plot respectively decision boundaries and drift rates as a function of DSR. Predicted drift-rates significantly increase as the target size decreases for both monkeys (resampling, *p* < 0.001), as might be expected from the shorter saccadic latencies associated with smaller targets. The predicted height of the decision boundary also increases significantly as the target size decreases for both monkeys (resampling, *p* < 0.001). This effect is more surprising as an increased decision boundary leads to longer saccadic latencies.

Therefore, modeling saccadic decisions as a stochastic accumulation leads to the (perhaps counterintuitive) prediction that smaller targets are associated with a higher accumulation rate and increased decision boundary, with a stronger increase in the accumulation rate leading to an overall drop in saccadic latencies. Note that this modeling (as well as in Harwood et al.^28^) relies on the assumption of a fixed noise level. Relaxing this assumption leads to an under-specified problem where these effects could be explained by any combination of joint changes in accumulation rate, decisional threshold, and noise level.

### Visual and motor peak activity

We then investigated how neuronal activity compares with behavior and predictions from our modeling framework. We first characterized cell activity in a delayed-saccade task (see example cells in Figure S6). All cells in both monkeys showed a significant increase of activity in the 100 ms around saccade-onset as compared to the 100 ms prior to target-onset (resampling, see STAR Methods, *p* < 0.05). Most cells (48/51 for Monkey Q and 24/28 for Monkey P) also exhibited a significant increase of activity in the 100 ms after target onset compared to 100 ms prior to target onset (resampling, *p* < 0.05). As almost all cells exhibited both visual- and saccade-related activity, we computed a Visuo-Motor Index (VMI)^34^ (see STAR Methods) to place cells on a continuum between pure visual cells (VMI of +1) to pure motor cell (VMI of −1). All cells except one (see Figure S7) had a VMI lower than 0, indicating that cells' activity in our sample was largely dominated by saccade-related responses.

**Figure 2:**
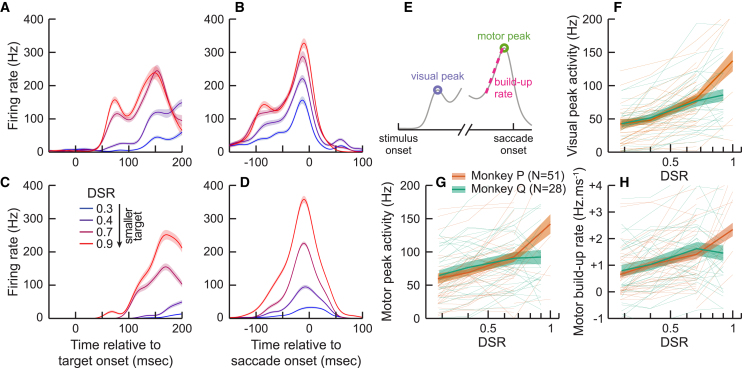
Visual and motor peak activity (A) Example of a neuron’s mean firing rate (line) and standard error (shaded area) as a function of time relative to stimulus onset (abscissa) and target DSR (colors). (B) Same as a function of time relative to saccade onset. (C) Same as (A) for a different neuron. (D) Same as (B) for a different neuron. (E) Depiction of the visual peak, build-up rate and motor peak. (F) Visual peak as a function of DSR (abscissa) for both animals (color). Thin lines are individual sessions. Thick line and shaded areas are the mean and SE. (G) Motor peak as a function of DSR. (H) Build-up rate as a function of DSR.

We then examined cell activity during the Ring task. Because the spread of saccadic endpoints was higher for larger rings than smaller rings, we only analyzed trials where saccade endpoints were within 2 degrees of the target center. Figures 2A and 2C plot the firing rate of 2 example cells as a function of target onset. The cell in Figure 2A shows a clear visual response approximately 50 ms after target onset for the 2 smaller target sizes (DSRs of 0.7 and 0.9). However, this visual response is almost non-existent for the 2 larger target sizes (DSRs of 0.3 and 0.4). The cell in Figure 2C exhibits a small visual response for the highest DSR and no visual response in other conditions. Figures 2B and 2D show the firing rate of the same 2 cells as a function of time relative to saccade onset. Both cells exhibit a sharp increase in firing rate in the 50 ms prior to saccade onset and peak shortly before initiation of the eye-movement, a typical pattern for cells in the superior colliculus.^35^^,^^36^ Both cells show a clearly reduced motor burst for larger targets than smaller targets, even though only trials with similar saccadic endpoints were analyzed. To quantify this effect, for each cell and condition we estimated peak visual activity (maximum firing rate in the 25–75 ms window after target onset), peak motor activity (maximum firing rate in the 50 ms preceding saccade onset) and motor build-up rate (increase in rate during the [-40,-10] ms window before saccade onset). These values are plotted in Figures 2F, 2G, and 2H, respectively. Comparison between the largest and smallest targets reveals an increase from approximately 40 Hz to 100 Hz in peak visual activity, while peak motor activity increased from approximately 70 to 150 Hz. Finally, the change in build-up rate increased from approximately 0.75 to 2 Hz ms^−1^. To test significance, we resampled our dataset and regressed peaks and build-up rates as a function of DSR for each sample. This analysis confirmed that peak visual activity, peak motor activity, and motor build-up rate all increased significantly for both monkeys as a function of DSR (resampling, *p* < 0.001).

### Relation between neural activity and behavior

Therefore, as predicted by our model, both peak motor activity and build-up rate increased when the targets became smaller. While this normative approach does not make predictions about visual responses, these strong modulations, comparable in amplitude to the change in motor peak activity, are potentially the source of the size-latency effect at a mechanistic level.

**Figure 3:**
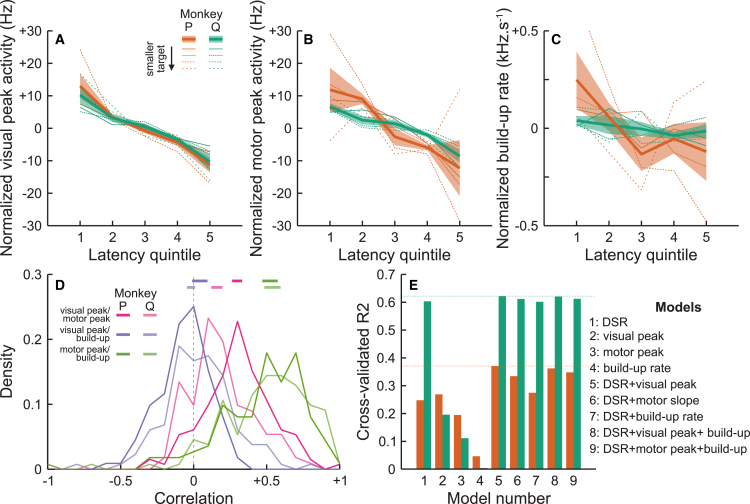
Relation between neural activity and behavior (A) Peak visual activity, normalized by the mean peak activity within condition, as a function of saccadic latency quintiles (abscissa) and stimulus condition (dashed lines) for both animals (green and orange). Shaded areas are standard errors across stimulus conditions. (B) Same for normalized peak motor activity. (C) Same for normalized motor build-up rates. (D) Distribution of correlation coefficients across trials, within cells and conditions, between visual peak and motor peak activity (pink), between visual peak and build-up rate (purple), and between motor peak and build-up rate (green). Thick horizontal lines at the top indicate 95% confidence interval of the distributions’ median. (E) Cross-validated R^2^ values of various generalized linear models.

To investigate the unmodeled visual transient modulations, we first looked at the relationship between neural activity and saccadic latencies within condition, that is, for an identical visual stimulus. We binned trials in quintiles of saccadic latency, and computed the same metrics for each latency bin. Figures 3A–3C plot peak visual activity, peak motor activity and build-up rate, respectively, normalized by the mean firing rate within condition and as a function of the latency bin. For both monkeys, peak visual and motor activity dropped by approximately 20 Hz between the fastest and slowest latency bins. The effect on motor build-up rate is less clear, with a trend towards a small decrease in build-up rate with increasing latency. We tested significance by resampling our dataset and regressing these metrics as a function of latency bins for each sample. Both visual peak activity and motor peak activity significantly decreased as latency increased for both animals (resampling, *p* < 0.001). However, the build-up rate did not significantly decrease for monkey Q (resampling, *p* = 0.16) and just reached significance for monkey P (*p* = 0.047). Consequently, even for identical visual stimuli a higher visual activity was associated with faster saccadic initiation times, and similarly for motor peak activity, while build-up rate seemed less clearly linked with saccadic initiation time.

Modulations in visual and motor activity at the scale of individual neurons in superior colliculus seem associated with substantial changes in saccadic latencies not only across conditions (the size-latency effect) but also within conditions (for identical visual stimuli). To make this link more explicit, we then investigated the relationship between visual and motor activity from trial to trial. For each trial, we estimated the peak visual activity, peak motor activity, and motor build-up rate, and computed their correlations within condition. These correlation factors, plotted in Figure 3D, were consistent across animals. There was a moderate correlation between visual peak and motor peak activity (median correlation 0.31 and 0.16 for monkeys P and Q, respectively). The median correlation was significantly higher than 0 for both animals (resampling of the distribution of correlation factors, *p* < 0.001). The correlation between visual peak activity and motor build-up rate was not significantly different than 0 for both animals (median correlation 0.04 and −0.05 for monkeys P and Q, respectively, *p* = 0.90 and 0.98). Finally, the correlation between motor build-up rate and motor peak activity was strongest and significantly different than 0 in both animals (0.54 and 0.53 for monkeys P and Q respectively, *p* < 0.001).

Finally, we tested whether saccadic latencies are better predicted by visual or motor activity. To address this question, we fitted and cross-validated various generalized linear models. The goal of this model comparison was to assess which variables are best predictors of saccadic latencies. Figure 3E shows cross-validated R^2^ values, a measurement of the variance explained by the model. Results are fairly consistent for both monkeys. Unsurprisingly, DSR alone explained a high amount of variance in saccadic latencies. Visual peak activity alone also explained a good amount of variance. These 2 variables together explained a little over 60% of the variance for one animal and almost 40% for the other, better than either variable alone. Remarkably, motor peak activity alone did not explain as much variance as visual peak activity, and the model including both DSR and motor peak activity did not explain more variance than the models including only visual peak activity. Finally, build-up rate alone explained very little variance in saccadic latencies, and including this variable in any model did not improve explained variance.

## Discussion

Here, we investigated the neural underpinning of the size-latency effect. Behaviorally, we found, as did prior studies,^28^^,^^29^^,^^30^^,^^31^ that saccadic latencies were strongly influenced by the Distance-to-Size Ratio (DSR). Stimuli in human studies consisted of a small ring target inside a large ring target, and observers were instructed to pay attention to either the small or the large target; the presence or absence of a central landmark target did not influence latencies, which depended instead on the attention scale condition.^28^ Here, to optimize neural data collection and remove the need for an additional attention task to constrain the animal’s attention, we displayed a single ring target on every trial, and the animals always performed the same eye-movement to the preferred saccadic location of the cell under study while target size was varied. Yet, the behavioral effect was remarkably similar, in range and amplitude, to the size-latency effect documented in humans. This finding demonstrates that although early investigations indicated an attentional role in the size-latency effect, the simple appearance of a large stimulus is sufficient to elicit it here (and in studies by De Vries et al.^30^ and Vullings et al.^31^). Overall, this confirms that the size-latency effect is well-suited for investigating simple saccadic decisions in primates.

We then fitted a stochastic accumulator decision model to the behavioral data. As in our prior study in humans,^28^ we predicted a higher accumulation rate and higher decision boundary with smaller targets (higher DSR). These joint effects are counterintuitive because they go in opposite directions, with a higher accumulation rate leading to faster saccadic latencies, and higher decision boundaries leading to slower saccadic latencies.

We compared these predictions with the actual firing rates of cells in the intermediate layers of SC that exhibit saccade-related activity.^22^ As predicted from modeling, we found that SC neurons have a higher build-up rate prior to saccade initiation as well as higher peak motor activity for higher values of DSR. This result is interesting because the decisional threshold and accumulation rate predicted by the normative stochastic accumulation model are theoretical constructs that can algorithmically be instantiated in different ways.^37^ Here, it seems that in the SC these theoretical variables are represented in a near direct fashion by the firing rates of individual neurons. Similar representations in the cortex are often more complex (e.g., Ditterich^38^ and Heitz and Schall^39^), perhaps reflecting the relative simplicity of SC due to its longer evolutionary history^40^ and relative proximity to the final motor output.^41^^,^^42^^,^^43^^,^^44^ However, despite a doubling of the motor peak activity, the velocity of saccadic eye-movements was relatively stable, and even dropped slightly for smaller targets in one animal. This result is surprising because saccadic latencies, spread and velocities have typically been observed to covary with motor activity of SC cells^45^^,^^46^^,^^47^ (but see Baumann et al.^48^). Overall, our results suggest that predictions from stochastic accumulator decision-models can be interpreted as a reasonable proxy for saccade-related activity in SC. This is potentially useful, for instance, for studies trying to explain cortical activity based on putative corollary discharges originating in SC (e.g., Wang et al.^49^).

We also found that visual activity was strongly modulated by the target’s size, with visual activity often being almost completely suppressed for the largest ring stimuli. This suppression was to be expected since cells in SC have suppressive surrounds, unlike antagonistic surrounds in other visual areas.^36^ We looked at the relationship between visual and motor activity with saccadic latencies not only across conditions (for different ring sizes) but also within conditions (for identical visual stimuli). We found that even within conditions, higher visual and motor peak activity were associated with shorter saccadic latencies. This result is in contrast with other studies that found a fixed level of activity at the time a motor response is produced both in the cortex^50^^,^^51^ and in SC.^52^^,^^53^^,^^54^^,^^55^ This observation is strengthened, first, by the fact that peak motor activity was correlated with peak visual activity, but not motor build-up rate, and second, from the fact that including build-up rates in a generalized linear model did not improve predictions from the model. The strong modulations observed here, with a doubling of peak motor activity between the smallest and largest DSRs demonstrates that reaching a fixed level of motor activity^54^^,^^56^^,^^57^ to trigger a saccade is not a general rule in SC (see also Baumann et al.^48^ and Edelman and Goldberg^58^), a finding that has also been observed in cortex.^59^

Taken together, these results suggest that the size-latency effect is a direct consequence of the visual responses of cells in SC. Larger targets elicit reduced visual activity over a larger area. This visual activity is followed by a fixed ramp-like increase of firing rate peaking shortly prior to saccade onset. Interestingly, the build-up rate was modulated by the target size, but uncorrelated with visual activity within condition. However, the build-up rate was highly correlated with motor peak activity. Cells in SC directly inhibit omnipause neurons,^60^ which, in turn, gate the activity of burst neurons in the brainstem. Saccadic velocities are directly related to the magnitude of hyperpolarization of omnipause neurons.^61^ We can conclude from the relatively constant saccadic velocities that the overall number of spikes sent to omnipause neurons was also relatively constant across conditions, despite the strong drop in motor peak activity for larger targets. However, we can only speculate about the relationship between build-up rate and DSR. Although the build-up of activity prior to saccade onset is usually modeled as linear for simplicity (e.g.,^50^^,^^51^^,^^54^^,^^55^^,^^62^), it actually tends to increase non-linearly, likely a result of lateral interactions between SC neurons.^47^^,^^48^ This would explain why both peak motor activity and build-up rates are reduced for larger targets.

Our results should be interpreted in the light of possible caveats. First, the distribution of recorded cells was heavily biased toward visuo-motor cells. We only selected cells that exhibited saccade-related activity and consequently, we did not observe the visual response of visual-only cells. However, it stands to reason that their visual responses would be comparable. Second, the visual peak was measured over a fixed 50 ms window during the first 75 ms after target onset. Faster saccade latencies are correlated with higher peaks, not shorter periods of visual activity. Although this time window is typically when visual target selection is thought to take place for simple stimuli such as this, our data do not by themselves imply that faster latencies are due to faster visual processing. Third, the build-up rate is measured over a smaller 30 ms window shortly before the saccade onset, which naturally only captures a snapshot of the build-up accumulation rate.

Although visual and motor activity in SC were originally thought to be independent,^63^ because microstimulation studies found no effect of stimulation parameters on eye movements^64^^,^^65^ (but see Stanford et al.^66^), it has since become clear that SC visual responses are a clear determinant of saccadic latencies.^58^^,^^67^^,^^68^^,^^69^^,^^70^^,^^71^^,^^72^^,^^73^ What is remarkable about the size-latency effect is the strength of these modulations. Previous studies reported clear but relatively small modulations of visual peak activity (few tens of Hz), associated with small changes in saccadic latencies (few tens of ms). Here, we observed dramatic modulations with firing rates sometimes dropping by hundreds of Hz (see Figures 2B–2D, and 2G), modulations that are seldom observed in physiological studies; and are associated with reliable changes in saccadic latencies of hundreds of ms (see Figure 1B). This result is consistent with the idea that the suppressive surround of SC cells is largely responsible for the size-latency effect—rather than an extended fixation zone,^74^ competition between populations of neurons,^19^ or local landmarks^58^^,^^72^—since a large ring is the stimulus that maximally stimulates a cell’s surround. To be clear, the cells’ motor responses are what trigger the saccadic eye movements, not the visual responses; and the motor responses fluctuate accordingly with predictions from a stochastic accumulator decision model. But from the observations that the size-latency effect occurs even though the monkeys performed the same eye-movements across trials, from the covariation of saccadic latencies with visual responses across and within conditions, and from the correlation between visual and motor responses, we conclude that the visual responses strongly, if not completely, influence the subsequent motor responses. This interpretation allows making clear and testable predictions. For a start, we can infer that this effect should not be limited to large ring targets like we used here, but should be elicited by any stimulus stimulating the surround of the cells encoding an upcoming saccadic eye-movement. One such stimulus is the remote distractor effect, where an irrelevant distractor is flashed during saccadic preparation and delays the triggering of the saccade. It seems likely that the cells’ suppressive surrounds are responsible for this effect, in a similar manner as the ring stimuli used here (an observation already pointed out by Olivier et al.^75^).

Our data provide a mechanistic explanation for how the size-latency effect is implemented in SC, but does not explain the functional significance of this effect. One possibility is that saccadic initiation time reflects, at least in part, the countermanding of ongoing saccade plans.^76^ Another possibility is that it reflects a cost-benefit tradeoff,^28^^,^^31^ similar to what has been reported for saccadic amplitudes.^77^ Finally, we should note that if the size-latency effect is caused by the visual response of cells in SC, as suggested by our results, this effect is unlikely to be fully accounted for by the SC alone. Studies in humans^28^^,^^29^ used stimuli consisting of a small ring target inside a larger ring target, and observers were instructed to pay attention to the small or large target. In the absence of an attentional modulation, this stimulus should elicit the same visual response in SC. Yet our results predict that the visual response would instead depend on which part of the stimulus observers pay attention to, suggesting that this task may be well suited for investigating attentional gating of visual signals by the cortex to SC.

### Limitations of the study

Here, we modeled saccadic decision-making using a normative, but simplistic, stochastic accumulator model. Such models typically fail to capture more complex decision strategies involving multiple processes (e.g., Heitz and Schall^39^).

Our study does not establish a causal role of the visual responses of SC neurons to the size-latency effect. Further investigation, including deactivation and microstimulation, would be required to establish this causal role.

## Resource availability

### Lead contact

Further information and requests for resources and reagents should be directed to and will be fulfilled by the lead contact, Baptiste Caziot (caziot@physik.uni-marburg.de).

### Materials availability

This study did not generate new unique reagents.

### Data and code availability

• The dataset supporting this study has been deposited on OSF. The DOI is listed in the key resources table.
• Code: All code supporting this study is available from the lead contact upon request.
• Any additional information required to reanalyze the data reported in this paper is available from the lead contact upon request.

## Acknowledgments

This research was supported by a National Science Foundation grant (BCS-1232654) to M.R.H., a National Institutes of Health grant (R01-EY030669) to R.M.M., and a Deutsche Forschungsgemeinschaft grant (524696675) to B. Caziot. We thank Junghyun Park, PhD for expert assistance with training and data collection.

## Author contributions

Conceptualization, M.R.H. and R.M.M.; data collection B. Caziot, B. Cooper, and R.M.M.; analysis B. Caziot; writing, review and editing, B. Caziot, M.R.H., and R.M.M.

## Declaration of interests

The authors declare no competing interests.

## STAR★Methods

### Key resources table

**Table:** 

REAGENT or RESOURCE	SOURCE	IDENTIFIER
**Deposited data**		

Raw Data	This study	https://doi.org/10.17605/OSF.IO/3VXTW

**Software and algorithms**		

MATLAB	Mathworks	R2021b
Custom Code	This study	N/A

### Experimental model and study participant details

All procedures were reviewed and approved by the Institutional Animal Care and Use Committee of State University of New York College of Optometry (Protocol RM2021-12-1), in accordance with the U.S. Public Health Service Policy on humane care and use of laboratory animals. A head-holder system and stainless-steel recording chamber to access the SC bilaterally were implanted under isoflurane anesthesia and aseptic surgical conditions in two male rhesus monkeys (Macaca mulatta; monkey P 8 years, ∼6 kg and monkey Q 11 years, ∼11 kg). Antibiotics (cefazolin sodium) and analgesics (buprenorphine hydrochloride) were administered as needed during the recovery period under the direction of a veterinarian. During recording periods, animals would follow a controlled water intake and were encouraged to participate in the task through liquid rewards, ensuring levels that neither impact their physiological health nor affect their psychological well-being.^78^

### Method details

#### Behavioral apparatus

Testing was performed in a dimly illuminated room. Experimental control, data acquisition, and presentation of visual displays were carried out by a custom real-time MATLAB program on a Macintosh G4 computer using the Psychophysics Toolbox.^79^^,^^80^^,^^81^ Visual stimuli were presented on a monitor positioned 29 cm in front of the monkeys. The monitor had a spatial resolution of 800 × 600 pixels and a refresh rate of 75 Hz. Eye position was sampled at 1 kHz using an EyeLink 1000 infrared video tracker (SR Research, Ottawa, Canada). Neural signals were amplified and bandpass filtered, and single units were isolated based on visual inspection of the activity waveforms and saved for offline analysis using Plexon’s MAP system (Plexon Inc, Dallas, TX, USA). Isolation was verified offline using the Plexon Offline Sorter (Plexon Inc, Dallas, TX, USA).

#### Procedure

In experimental sessions, a microelectrode with an impedance of 1–2 MΩ at 1 kHz (FHC Inc, Bowdoin, ME, USA) was lowered using a stainless-steel guide-tube to locations in the SC which had been previously mapped. Once a cell was well isolated, its preferred saccade direction and amplitude were estimated in a delayed-saccade task by manually varying the location of the target across trials. Only cells showing saccade-related activity in the delayed-saccade task were recorded in the subsequent Ring Task. The average number of trials collected per session for the Ring Task was 400 for monkey P and 396 for monkey Q.

#### Delayed-saccade task

A white square fixation point subtending 0.25° with a luminance of 1.5 cd/m^2^ appeared in the central position against a homogenous dim background of 0.2 cd/m^2^. Monkeys were required to keep their eyes within a 1–1.5° window around the fixation point during an initial fixation interval of 450–650 ms. At the end of this interval, the fixation point remained illuminated and a target stimulus was presented at a peripheral location aligned with the RF of the cell. Monkeys were required to maintain fixation until the disappearance of the fixation point approximately 500 ms later. Once the fixation point disappeared, the monkeys were rewarded for making a saccade to the peripheral stimulus. Eye-position tolerance windows around the target (area where the saccadic endpoint was required to land for the trial to be rewarded) were equal to the stimulus eccentricity divided by 5. The increase in window size with eccentricity was chosen from experience to accommodate the higher spread of saccadic endpoints with eccentricity, such that the animal received rewards at an approximately constant rate across conditions.

#### Ring task

Once we located the center of a cell’s movement field, we presented ring stimuli centered at the cell’s preferred location. We varied the size of the rings to produce varying values of Distance-to-Size Ratio (DSR). The rings had a luminance of 12.4 cd/m^2^ and a thickness equal to the eccentricity of the ring center divided by 20, and were presented on background of 0.2 cd/m^2^. The fixation mark disappeared and the target appeared approximately 500 ms after fixation onset. Ring targets were used for comparison to the existing human studies and to avoid the addition of any attentional constraint task, which would be required if a discrete central reference point was provided because otherwise the outer target ring could simply be ignored by the monkey. The presence or absence of a central landmark target has been shown not to influence latencies, which are determined by the size of the attended target region.^28^ Monkeys were rewarded for making a saccade to the center of the ring. The eye-position tolerance window around the ring center was equal, as for the delayed-saccade task, to the eccentricity of the ring center divided by 5. For monkey Q, DSR values were always 0.29, 0.40, 0.67 and 0.90. For monkey P they were 0.29, 0.40, 0.67 and ≥1 (DSR values higher than 1 were used during training). Because the Distance-to-Size latency effect saturates at a DSR value of 1,^28^ we pooled all DSR values higher than 1 and plot them as 1 in Figures for clarity.

#### Cell classification

We characterized the relative strength of the visual and movement-related responses of the cells with a Visuo-Motor Index (VMI^34^) calculated using data from the delayed-saccade task. First, we estimated whether each cell had saccade-related activity by comparing activity in the 100 ms prior to saccade initiation to 100 ms prior to target onset. Second, we estimated whether cells had a visual response by comparing activity in the 100 ms range after target onset to the 100 ms prior to target onset. Significance was established by resampling 1,000,000 times the difference in mean firing-rate between 2 event windows. VMI was then computed as:

$$VMI=\frac{V-M}{V+M}$$

where V and M are the peak visual (V) and motor (M) activity in the aforementioned windows. This index varies from −1 to +1, with −1 indicating a cell that has only a motor response, and +1 a cell that has only a visual response, and 0 a cell with visual and motor responses that are equal in magnitude. The goal of this index is to capture the continuum between so-called visual neurons, motor neurons and visuo-motor neurons.

### Quantification and statistical analysis

Offline data analysis was performed using custom MATLAB (Mathworks) scripts. Saccade onset was estimated as the first time at which eye velocity exceeded 55 deg.s^−1^. To generate continuous spike-density functions, neural events were convolved with an acausal Gaussian filter (σ = 5 ms). For statistical reliability, only conditions having at least 10 trials were included in the analyses (84% and 100% of conditions for monkeys P and Q respectively). For physiological analyses, only trials with saccadic endpoints falling within 2 deg of the cell’s preferred saccadic location were analyzed (31, 50, 50 and 68% of trials for increasing DSR for monkey P, and 30, 58, 93 and 98% for monkey Q). When assessing statistical significance, we adopted a criterion α-level of 0.05. Unless specified otherwise, statistical testing was done by resampling, either individual trials within a session or the mean across sessions, 1,000,000 times, and re-estimating the tested parameter for each sample (e.g., the slope parameter for a linear regression). Unless otherwise specified, all tests were two-sided.

For model comparison, for each cell separately we randomly selected 20% of the dataset as a validation set. We fitted a GLM to the remaining 80% of the data, then computed the adjusted R^2^ values of the model on the validation set, according to:

$$R^{2}=1-\frac{\sum {\left(y_{i}-\hat{y_{i}}\right)}^{2}}{\sum {\left(y_{i}-\bar{y}\right)}^{2}}.\frac{n-1}{n-p-1}$$

With n and p the number of observations and number of model parameters respectively. We performed this comparison 1,000,000 times and computed the mean R^2^ value across cells and samples. We regressed inverse saccadic latencies with DSR values as categorical variables (4 groups), peak visual activity, peak motor activity and motor build-up rate, by finding the regressor weights β that minimize the following equation:

$$\min_{\beta }\;\sum_{i}{\left[\left(\sum_{j}\beta_{j}.X_{j}\right)-\frac{1}{Y_{i}}\;\right]}^{2}$$

With $X_{j}$ and $\beta_{j}$ the value and weight of the regressor j, and $Y_{i}$ the saccadic latency of the trial i.

#### Accumulator model

We modeled decisions as the accumulation of a noisy signal toward a decision boundary. A saccade is initiated when the decision boundary is crossed. We assumed that the accumulated signal is generated by a Wiener process with drift μ and variance $\sigma^{2}$

dx=μdt+σdW

Therefore, the probability for the accumulator to reach the decision bound z at a time t follows an inverse Gaussian distribution^82^:

$$f\left(t|z,\mu ,\sigma \right)=\frac{z}{\sigma \sqrt{2\pi t^{3}}}e^{\frac{-{\left(z-\mu t\right)}^{2}}{2\sigma^{2}t}}$$

We used Maximum Likelihood Estimation to fit the distribution of saccadic latencies with the predicted distribution:

$$\underset{\theta }{\mathrm{argmax}}\sum_{i}\log\left(f\left(t_{i}|\theta \right)\right)$$

For comparison, we also fitted a LATER saccadic decision model.^62^ Results across models were highly correlated (see Figure S4) and all results described here remain valid using either model.
