# Supplementary material for: Physiological correlates of a simple saccadic-decision task to extended objects in superior colliculus
Source: iScience. 2025 Jul 23;28(8):113179. doi: 10.1016/j.isci.2025.113179 (PMC12432453; doi:10.1016/j.isci.2025.113179)
Supplement: Document S1. Figures S1–S7 [file mmc1.pdf]

iScience, Volume *28*

Supplemental information

Physiological correlates of a simple saccadic-decision task to extended objects in superior colliculus

Baptiste Caziot, Bonnie Cooper, Mark R. Harwood, and Robert M. McPeek

SUPPLEMENTAL INFORMATION

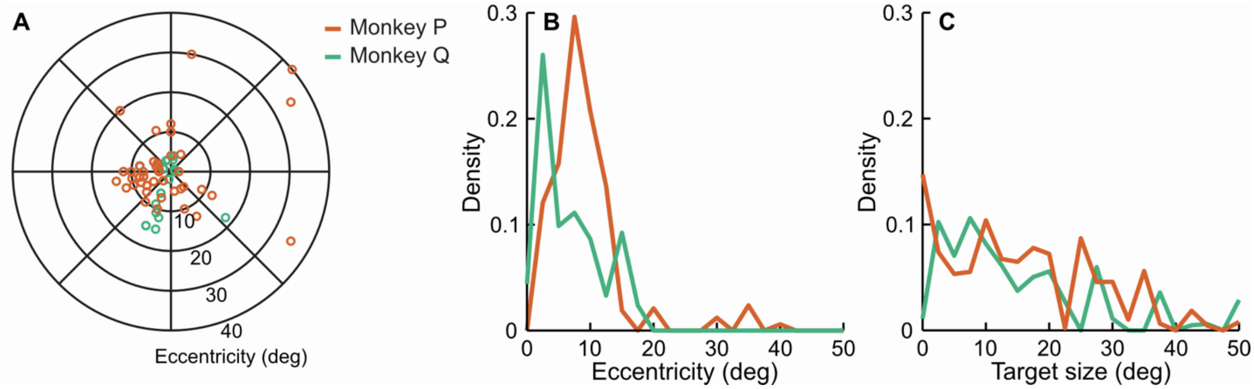

Figure S1: (A) Preferred saccadic location of all recorded cells for both monkeys (orange and green). (B) Distribution of target eccentricity. (C) Distribution of target sizes.

Figure S1A plots the preferred saccadic location of all cells for monkey P (51 cells) and Q (28 cells). Figure S1B plots the distribution of target eccentricity across all trials for both monkeys. Targets were always centered on the cell's preferred saccadic location. Figure S1C plots the distribution of target sizes. Target sizes were calculated as a function of target eccentricity to yield discrete 4 DSR values as:

DSR = Distance / Size

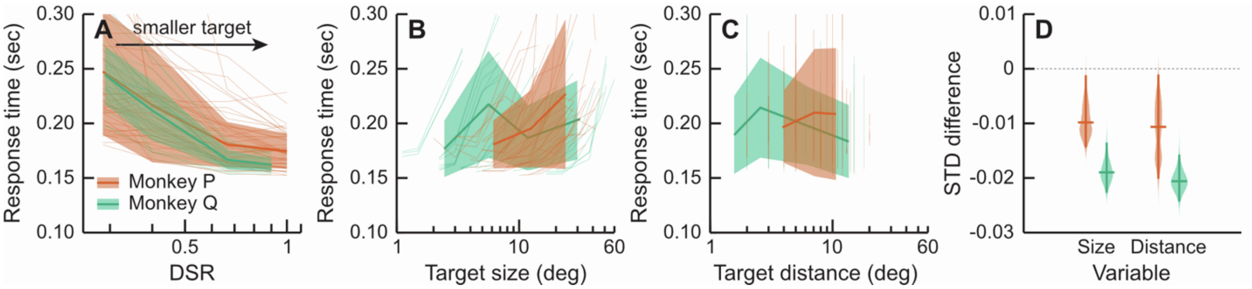

Figure S2: (A) Same as Figure 1B: median saccadic latency as a function of DSR for both animals (green and orange). Thin lines are individual sessions, thick lines mean across sessions and shaded area standard deviations (not standard errors as in Fig. 1B). (B) Same as A but plotted as a function of target size (abscissa). (C) Same as A but plotted as a function of target distance (abscissa). (D) Difference between the standard deviation within 4 bins when saccadic latencies are grouped by DSR as compared to when they are grouped by Target Size or Target Distance.

Saccadic latencies are better explained by the target Distance to Size Ratio (DSR), rather than just Target Size or Target Distance (eccentricity). Figure S2A plots saccadic latencies as a function of DSR, Figure S2B as a function of Target Size and Figure S2C as a function of Target Distance. Each line corresponds to a different recording session where target eccentricity (and therefore saccadic amplitude) was fixed for the duration of the session but could vary across sessions. DSR within each session was consequently directly related to Target Size. To estimate if saccadic latencies were better explained by DSR than Target Size or Target Distance, we binned median latencies in 4 bins as a function of either DSR or Target Size or Target Distance and computed the mean standard deviation of median latencies across bins. For both animals the standard deviation of median latencies was smaller when binned by DSR as compared to either Target Size or Target Distance. This demonstrates that DSR, the ratio of the target distance (eccentricity) over its size is a better descriptor of saccadic latency than either Size or Distance alone.

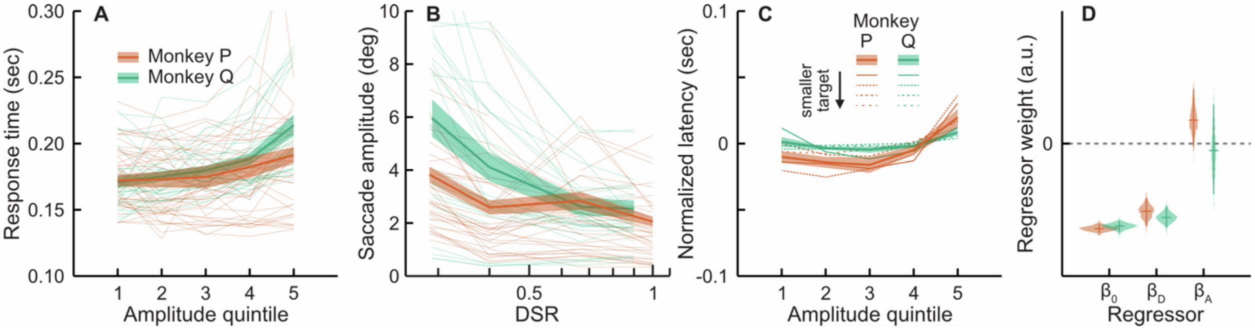

Figure S3: (A) Median saccadic latency (ordinates) as a function of saccadic amplitude quintile (abscissa) for both animals (green and orange). Thin lines correspond to individual sessions, thick lines to mean across sessions and shaded area to standard errors. (B) Median saccadic amplitude (ordinates) as a function of DSR for both animals (green and orange). Thin lines correspond to individual sessions, thick lines to mean across sessions and shaded area to standard errors. (C) Median saccadic latency (ordinates), normalized by the median latency within DSR condition, as a function of saccadic amplitude quintile (abscissa) and stimulus condition (dashed lines) for both animals (green and orange). Shaded areas are standard errors across stimulus conditions. (D) Multiple-variables fit parameters (abscissa) for both animals (green and orange). Horizontal and vertical lines are medians and 95% confidence intervals of the distributions.

Saccadic latencies are *positively* correlated with saccadic amplitudes for both monkeys (median ρ within sessions=0.10 and 0.35, ρ significantly different that 0 in 23/51 and 18/28 recordings for monkeys P and Q respectively). Figure S3A plots saccadic latency as a function of saccadic amplitude and shows that saccadic latencies tend to increase slightly with saccadic amplitudes. However, saccadic amplitudes are also correlated with stimulus conditions (DSR). Figure 3B shows the relationship between DSR and saccadic amplitudes. Figure S3C shows saccadic latencies as a function of saccadic amplitude quintile for each stimulus condition separately, normalized by the median saccadic latency within condition. Saccadic latencies appear relatively unmodulated by saccadic amplitude once the modulation introduced by DSR is taken into account. To assess significance, we used a multiple-variable linear regression. Since saccadic latency decreases approximately exponentially with both DSR and amplitude, we regressed log latencies with DSR and amplitude: $\log(L) = \beta_0 + \beta_D . D + \beta_A . A$ with L the saccadic latency, D the DSR condition, and A the saccadic amplitude. We resampled the dataset for each monkey separately and ran the regression on each sample. Figure S3D plots the distribution of regression parameters. The weights of both the intercept (β_0) and DSR condition are significantly lower that 0 (p<0.001). The weight of the saccadic amplitude is not significantly different than 0 for either monkey (p=0.08 and 0.39). This demonstrates that the reduction in saccadic latencies with DSR condition is not caused by an increase in saccadic amplitude.

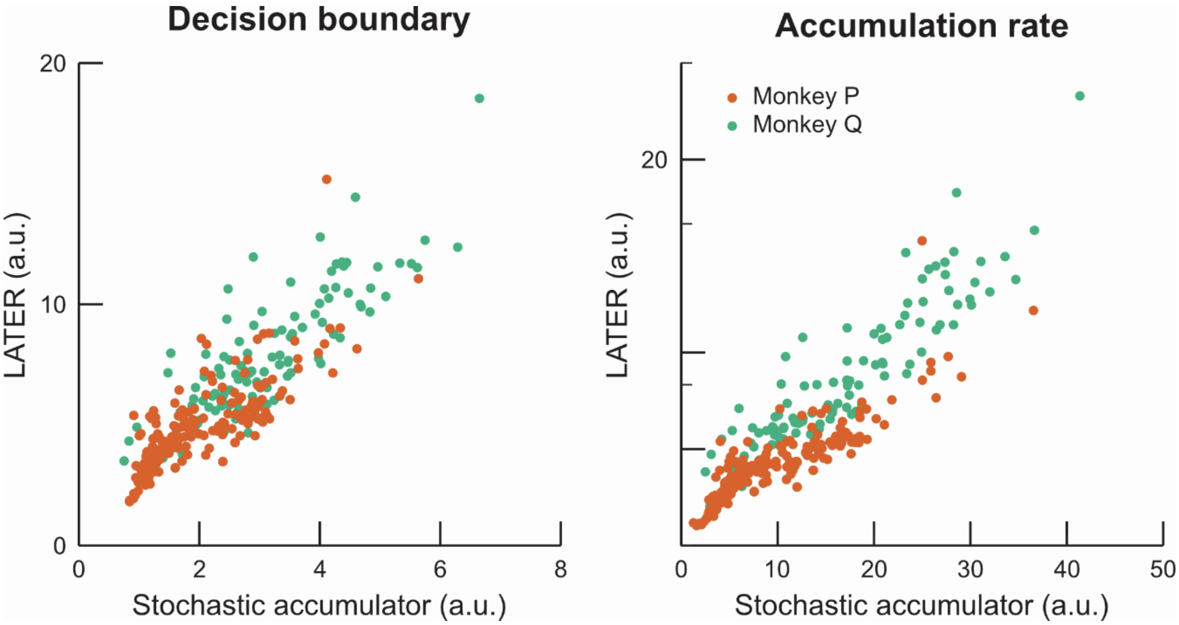

Figure S4: Left: Decision boundaries predicted from fitting the LATER model as a function of decision boundaries predicted from the stochastic accumulator model for both animals (colors). Right: Same for accumulation rates.

For each session we fitted a stochastic accumulator model and a LATER model [28,58]. Both models assume the accumulation of a signal towards a decision boundary. The LATER model predicts a reci-normal distribution and the stochastic accumulator predicts an inverse-Gaussian distribution, which are very similar. Indeed, parameters predicted from either model were highly correlated: 0.91 and 0.92 for the accumulation rate for monkeys P and Q respectively; 0.81 and 0.85 for the decision boundary. All results described in the main text remain valid using either model.

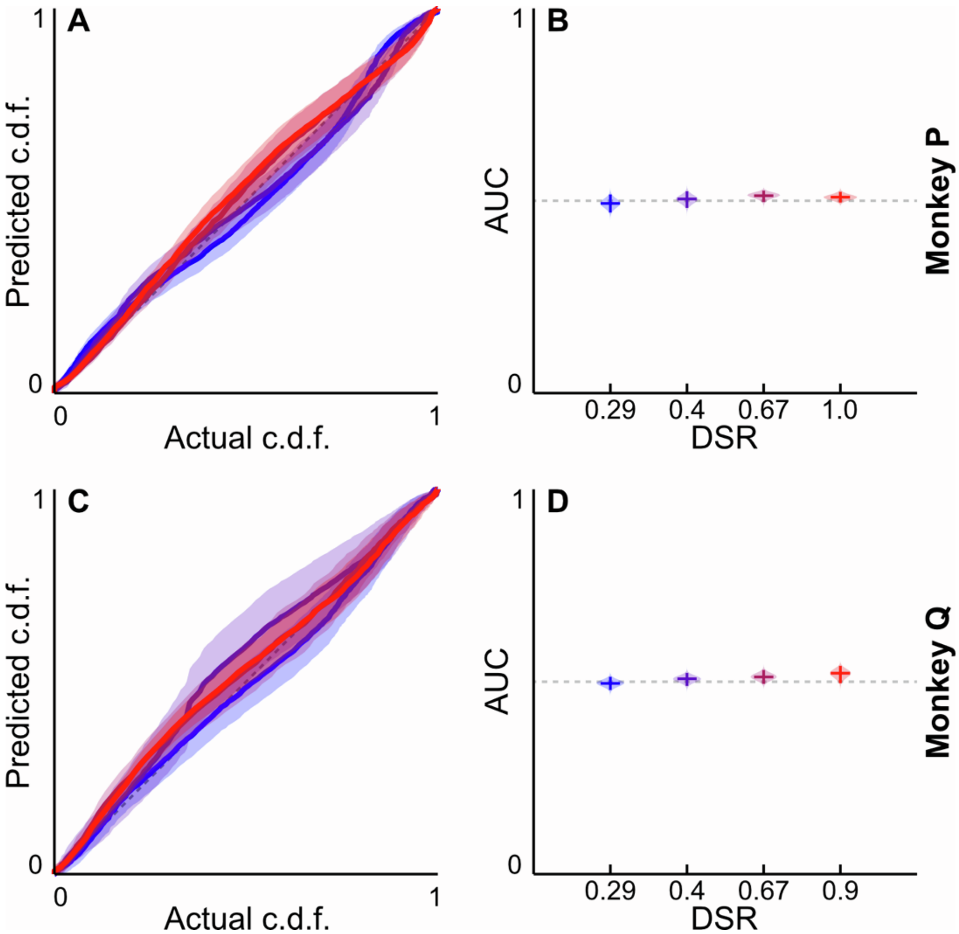

Figure S5: (A) Quantile-Quantile plot of the mean cumulative density function of saccadic latencies predicted by the model (ordinates) as a function of the mean actual cumulative density function (abscissa) for each stimulus condition separately (colors) and for monkey P. Shaded areas are 95% confidence interval. (B) Area Under the Curve (AUC, ordinates) of the QQ plots as a function of DSR (abscissa). Shaded areas are violin plots and vertical bars are 95% confidence intervals. (C) Same as A for monkey Q. (D) Same as B for monkey Q.

We assessed goodness-of-fit of the stochastic decisional model by estimating the Area Under the Curve (AUC) of Quantile-Quantile plots of the cumulative distributions. In short, we plot each quantile of the predicted distribution of saccadic latencies as a function of the same quantile of the actual distribution of saccadic latencies. We then compute the AUC by integrating the area under the QQ plots. Identical distributions would produce an AUC value of 0.5. The AUC values were not significantly from 0.5 for any stimulus condition or animal.

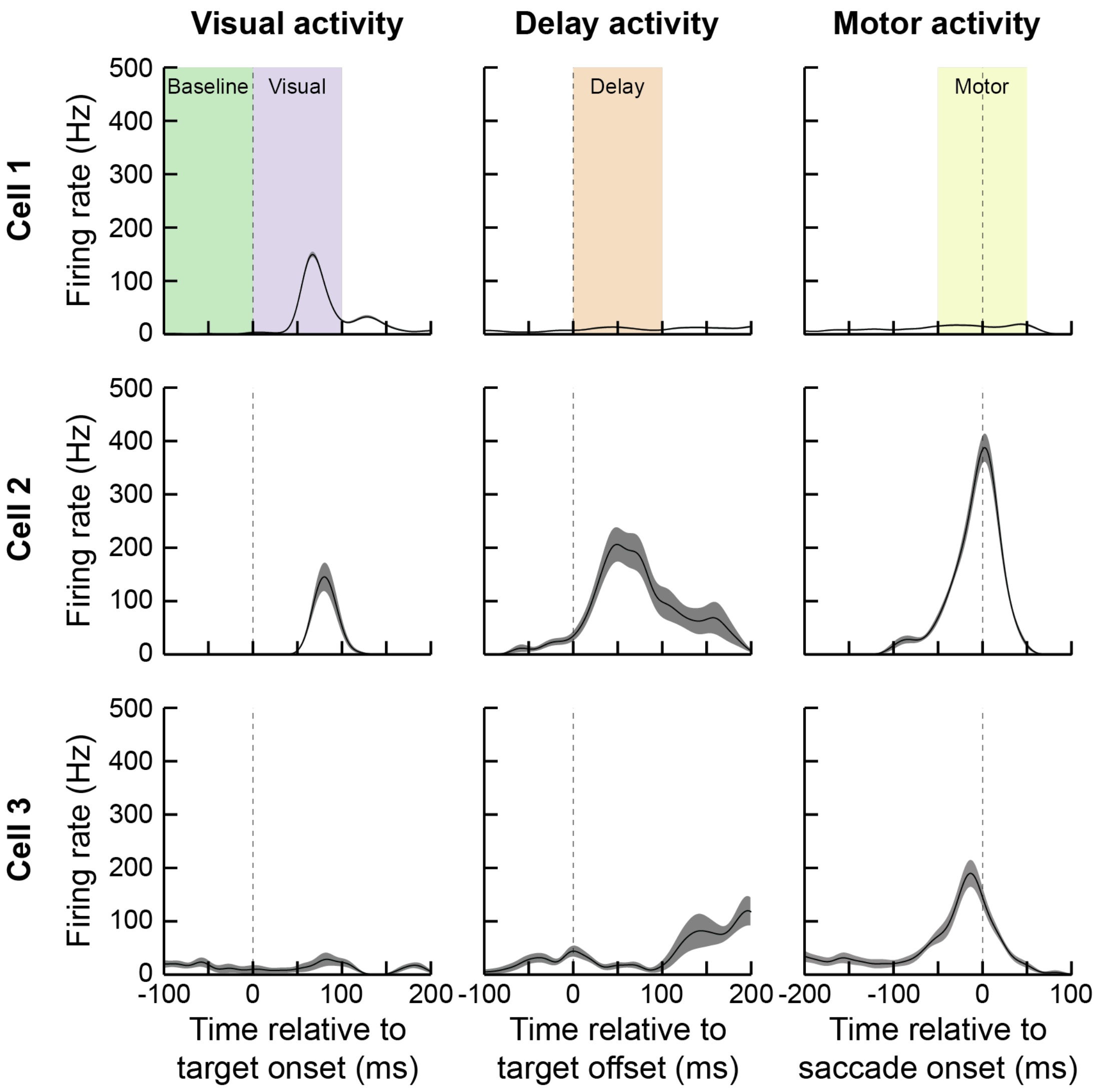

Figure S6: Example responses in the delayed saccade task for 3 neurons (rows). The left columns plots mean firing rate (black line) and standard error (shaded area) as a function of time relative to target onset. Middle column relative to target offset and right column relative to saccade onset.

We classified cells with a delayed-saccade task. This task allows dissociating visual from motor activity. Figure S6 shows 3 example cells. The first cell (top row) exhibits a clear increase in firing rate between 50 and 100ms after target onset (left graph), but no change in activity when the target disappears (middle) or when the animal performs the eye-movement (right). The second cell (middle row) also exhibits a clear visual response, as well as an increase in firing rate when the target disappears, and a strong increase in firing rate peaking approximately at eye-movement onset. The last row shows a cell that has only motor activity.

To test whether a cell exhibited visual, delay or motor activity, we computed the mean firing rate within 3 time-windows (shaded color areas in Figure S6) and compared this activity to the baseline activity of the neuron in the 100ms prior target onset.

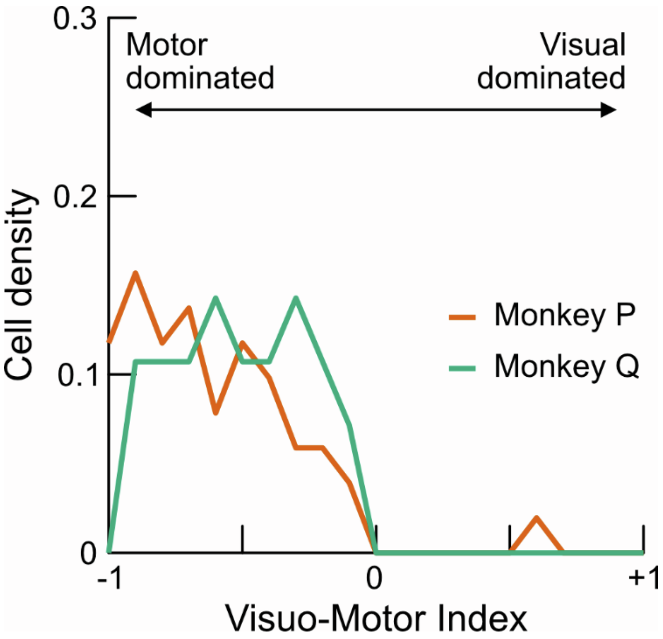

Figure S7: Histogram of Visual-Motor Indices for all cells of both animals (color). A value of -1 indicates a pure motor cell (clear motor response, no visual response) and a value of +1 indicates a pure visual cell (clear visual response, no motor response).
